# The Need for Evidence Based Nutritional Guidelines for Pediatric Acute Lymphoblastic Leukemia Patients: Acute and Long-Term Following Treatment

**DOI:** 10.3390/nu5114333

**Published:** 2013-10-31

**Authors:** Joyce L. Owens, Sheila J. Hanson, Jennifer A. McArthur, Theresa A. Mikhailov

**Affiliations:** Division of Critical Care, Department of Pediatrics, Medical College of Wisconsin/Children’s Hospital of Wisconsin, 9000 W. Wisconsin Avenue, M/S #681, Milwaukee, WI 53226, USA; E-Mails: shanson@mcw.edu (S.J.H.); jmcarthu@mcw.edu (J.A.M.); tmikhail@mcw.edu (T.A.M.)

**Keywords:** antioxidants and cancer, childhood leukemia, leukemia, nutrition, antioxidant supplementation in leukemia, childhood cancer

## Abstract

High survival rates for pediatric leukemia are very promising. With regard to treatment, children tend to be able to withstand a more aggressive treatment protocol than adults. The differences in both treatment modalities and outcomes between children and adults make extrapolation of adult studies to children inappropriate. The higher success is associated with a significant number of children experiencing nutrition-related adverse effects both in the short and long term after treatment. Specific treatment protocols have been shown to deplete nutrient levels, in particular antioxidants. The optimal nutrition prescription during, after and long-term following cancer treatment is unknown. This review article will provide an overview of the known physiologic processes of pediatric leukemia and how they contribute to the complexity of performing nutritional assessment in this population. It will also discuss known nutrition-related consequences, both short and long term in pediatric leukemia patients. Since specific antioxidants have been shown to be depleted as a consequence of therapy, the role of oxidative stress in the pediatric leukemia population will also be explored. More pediatric studies are needed to develop evidence based therapeutic interventions for nutritional complications of leukemia and its treatment.

## 1. Introduction

Pediatric acute lymphoblastic leukemia (ALL) is the most frequently occurring cancer in children and adolescents. An overview of the physiologic processes by which leukemia develops and potential nutritional implications will be reviewed. The treatment protocols used in pediatric oncology patients are more aggressive than those used in adults, with resulting differences in nutrition-related complications of leukemia and its treatment.

Current pediatric treatment protocols yield a cure rate approaching 90% [1]. This excellent cure rate means that there are and will be more childhood cancer survivors reaching adulthood. The higher cure rate obtained in children has not been duplicated in adolescents or older adults [2]. The ALL cure rate declines in adults to approximately 40%. In the United States (U.S.) sixty percent of children under the age of fifteen participate in clinical trials compared to less than 5% of adolescents and young adults. Improvement in pediatric survival rates has been attributed to early detection, enhanced treatment involving the use of multiple treatment modalities, management of infections and improvement in supportive care [3]. These advancements in care may be facilitated by the high rates of pediatric participation in study trials.

The substantial gains in pediatric oncology treatment have caused an array of adverse treatment related effects leading to an increase in the complexity of assessing acute and long-term nutritional status as well as nutritional needs. These adverse effects may have an acute or delayed onset [3]. One of the more commonly described nutrition-related adverse effects from chemotherapy is depletion of antioxidants. This is especially problematic during the conditioning phase as this is the most intense phase of treatment. During conditioning, patients receive high doses of chemotherapy without much break in between in order to place the patient into remission from their cancer. Many chemotherapeutic agents produce oxygen free radicals and deplete the body’s stores of antioxidants while attempting to neutralize them. In addition, patients typically feel quite ill during this phase and often are not able to tolerate and/or consume adequate nutritional intake, which makes it difficult to replenish their antioxidants. Other adverse effects of treatment include malnutrition, an increase in total body fat or obesity (especially in female ALL patients treated with cranial irradiation), and heightened protein turnover in children treated with high dose intravenous methotrexate and glucocorticoids [4]. An increased body mass index (BMI ≥ 25 kg/m^2^) has been exhibited in childhood survivors of ALL, whose age at the time of diagnosis was less than 4 years, [3,5,6,7]. Other adverse effects include several chronic conditions: endocrine disorders, hypothyroidism, disorders of growth and pubertal development, cardiovascular disease, pulmonary disease, neurological and neurosensory disorders, osteonecrosis, secondary cancers, fatigue, and chronic pain.

Currently, no uniform evidence based nutritional guidelines have been established addressing the optimal nutritional prescription regarding energy, physical activity, nutrients, and the use of dietary supplements during and after treatment [4]. This article will review and discuss the recent literature and highlight areas in which further research is needed.

## 2. Overview of Leukemia

Cancer is a disorder of cell growth and regulation, leading to abnormal cell division and production [8]. This dysregulation results in unlimited, dysfunctional cell production [9]. These unregulated cells may form cancer cells that then can spread throughout the body [8].

Leukemia is a form of cancer that originates in the bone marrow and is released into the blood stream [10]. The bone marrow is responsible for producing stem cells that will eventually mature into the different types of blood cells: red blood cells (RBC), white blood cells (WBC) and platelets. White blood cells are comprised of granulocytes (eosinophils, basophils, and neutrophils), monocytes (macrophages) and lymphocytes (B-cells and T-cells) [10]. Stem cells mature into two different myeloproginator cell lines, lymphoblasts and myeloblasts. Lymphoblasts mature into the type of WBC called lymphocytes. Myeloblasts differentiate into RBC, non-lymphoid WBC or platelets. Cancer cells produced from the myeloid or lymphoid cell lines by the bone marrow are termed leukemia cells. Leukemia is categorized as an acute (rapid onset and progression) or chronic (slow onset and progression) production of malignant blood cells. It is further sub-categorized according to the type of WBC affected [11]. White blood cells that are affected include lymphoid (lymphocytic or lymphoblastic) or myeloid cells (myeloid). The four most common types of leukemia include chronic lymphocytic leukemia, chronic myeloid leukemia, ALL, and acute myeloid leukemia [11]. Both ALL and acute myeloid leukemia commonly occur in children. The annual incidence of leukemia in the U.S. is 30 to 40 per one million. In children and adolescents under the age of 20, the annual incidence of ALL amounts to 2900 new cases per year in the U.S. The highest incidence of leukemia in children occurs between the ages of 2–3 years in the U.S. [12].

The development and function of WBC and their cellular differentiation requires nutritional factors. These nutritional factors include vitamins and minerals such as vitamin A, zinc, and iron. In particular, the B-vitamins (folate, cobalamin, niacin and pyridoxine) are critical for cellular deoxyribonucleic acid synthesis [10].

No one causative factor has been linked to cancer development. Childhood cancer most often involves transformation of stem cells and is the result of a spontaneous mutation in the area of the genes that control the growth and life cycle of the cells involved [1,13]. These mutations can develop at any of the numerous stages of normal lymphoid differentiation [14]. Genetic mutations can be detected in 75% to 80% of all childhood ALL. Signs and symptoms of leukemia are related to the quantity and location of leukemia cells in the body. Leukemia cells can affect many different body systems and organs such as the brain, kidneys, heart, lung, and gastrointestinal tract. Common signs and symptoms of pediatric acute and chronic leukemia include general malaise, gums that bruise or bleed readily, recurrent infections, enlarged lymph nodes, bone or joint pain, fevers, pallor and abdominal discomfort or swelling.

The diagnosis of leukemia involves a physical examination to assess for enlarged lymph nodes, liver, or spleen. A simple blood test, the complete blood count, is used to evaluate the number of blood cells (WBC, RBC and platelets). In leukemia there is an enhanced production of immature blast cells, but they are not capable of maturing into functional cells. The overproduction of one cell line in the bone marrow leads to an increased number of blast cells, crowding out the production of other normal cells such as RBC and platelets, often causing the patient to be anemic and thrombocytopenic. To confirm the presence of leukemic cells in the bone marrow, a bone marrow aspirate or biopsy is performed. Other tests are usually performed to determine the specific type of leukemia present through assessment of chromosome abnormalities (cytogenetics) and lumbar puncture to assess for the presence of leukemic cells in the cerebrospinal fluid [9].

There are several treatment options available for leukemia based on the individual’s age, leukemia type, and the location of leukemic cells. Major treatment options involve chemotherapy using a combination of drugs, radiation therapy (high-energy rays used to destroy cancer cells), biological therapy (drugs to enhance the body’s own defense against cancer) and hematopoietic stem cell transplantation (HSCT). The latter enables high doses of chemotherapy, radiation therapy, or both to be used. High dose therapy destroys both normal and leukemic cells. Once the high dose chemotherapy is completed, normal stem cells are administered as replacement for the blood cells destroyed by the treatment. Stem cells are acquired by self (patient) donation (autologous), family member or matched unrelated donor (allogeneic), or identical twin (syngeneic). This topic and other treatment modalities result in important nutritional consequences of cancer and will be further discussed.

## 3. Nutrition Considerations

Malnutrition is a general phrase that serves to define an inadequate nutritional state. Malnutrition ensues from an imbalance in consumption (insufficient or excessive), and utilization of energy, nutrients and/or both [15,16]. Malnutrition may be a consequence of the cancer itself and/or its treatment. The occurrence of malnutrition in pediatric oncology patients cited in the literature varies extensively, largely due to a lack of a consensus in identifying and classifying malnutrition. The World Health Organization defines malnutrition as a BMI less than the 5th percentile, a criterion, which most pediatric oncology patients do not meet [3]. There is also no consensus regarding the identification of nutritionally at risk children with cancer. A diminished nutritional status is a potential risk factor for reduced immune function, altered drug metabolism, leading to drug toxicities, and prolonged wound healing. Thus malnutrition has the potential to cause appreciable adverse clinical outcomes [3,10,16] and reduced quality of life and overall well-being [16].

Standard parameters used in assessing nutritional status are often altered in pediatric oncology patients. Corticosteroid-induced edema can mask undernutrition (weight gain as a result of fluid retention) thereby negating weight as an accurate nutritional marker of nutritional status. Weight may further be altered by hydration status during chemotherapy [3]. Lack of clear definitive guidelines for assessing or identifying children and young adult oncology patients with malnutrition or at risk for malnutrition, makes it difficult to obtain an accurate prevalence rate of malnutrition in this population [3,17], though the rate is approximated at 46% [3].

Serum protein markers have limited use in identification of malnutrition as their concentrations are affected by the liver’s rate of synthesis, degradation, and seepage from the circulatory system [15]. During inflammation or infections the synthesis of acute-phase proteins by the liver such as ceruloplasmin, C-reactive protein and ferritin is up-regulated while the synthesis of negative acute-phase proteins such as albumin, prealbumin, retinol-binding protein and transferrin is down-regulated [15] as a consequence of the stress response. Furthermore, chemotherapy and recurrent episodes of infection and sepsis may further diminish body nutrients (zinc in particular) as a result of altered metabolism leading to sub-optimal growth. An unrecognized depletion of micronutrients may also result as a consequence of decreased food consumption, significant gastrointestinal losses or increased nutrient requirements, further adding to the complexity of malnutrition in this population [3,10]. Weight is also not a precise representation of long-term alteration in body cell mass [3] related to several factors. Weight may be appropriate or excessive, yet loss of lean body mass has occurred. Fat mass may decrease or remain stable, even though wasting of skeletal muscle has occurred. The loss of cell mass (skeletal muscle) is associated with impaired immune and pulmonary function, increased disability and mortality [3,15]. 

A standardized definition and description of malnutrition and the predominant factors associated with pediatric malnutrition in the developed world has been proposed [18]. The proposed standardization classifies the cause of malnutrition into two broad categories, illness and non-illness related. Illness related causes are associated with disease (*i.e.*, cancer), trauma/injury, surgery and/or chronic conditions. Non-illness related causes are associated with behavioral and/or environmental causes leading to undernutrition. Illness related and non-illness causes are further divided into acute (onset less than 3 months ago) or chronic (onset 3 months ago or more) [18]. A homogeneous method of defining and classifying malnutrition criteria will allow for a more accurate identification and documentation of the incidence of malnutrition [18]. Establishment of malnutrition criteria may also facilitate the development of nutritional guidelines and rapid enactment of appropriate nutritional interventions.

## 4. Pathophysiology of Malnutrition in Children with Leukemia

Several pathophysiological processes lead to the development of growth failure and malnutrition in this population. These processes include inflammation, skeletal muscle breakdown, loss of body proteins, and lipid oxidation. Aggressive multimodal cancer therapy and its induced toxicities can result in altered gastrointestinal function, absorption, metabolism, and utilization as a consequence of altered hormonal response and metabolic demands. Pain (*i.e.*, mucositis) and disorders of appetite, dysgeusia and xerostomia can further increase the risk for inadequate nutritional intake leading to increased risk of malnutrition [3]. Body composition changes also affect the absorption, distribution, metabolism, and elimination of cytostatic medications. Opiate pain medications such as morphine decrease oral intake due to their propensity to cause nausea, constipation, and anorexia [10].

Other treatment complications related to nutrition involve nutrient-medication interactions. For example, methotrexate inhibits folate metabolism. Cyclosporine alters potassium and magnesium homeostasis, which can lead to depleted serum levels requiring nutritional replenishment. Glucocorticoid steroids induce hyperglycemia, fluid retention, weight gain (fat mass) resulting in altered body composition, electrolyte abnormalities, and increases requirements for calcium, zinc, and vitamins D and C with long term use [10,19]. A significant increase in long-term fat mass (altered body composition) has been reported in pediatric leukemia patients prescribed glucocorticoid steroids in conjunction with methotrexate [4].

The hormone leptin is the chief regulator of appetite and satiety. Elevated levels of leptin down-regulate appetite and enhance energy utilization [20]. Human studies in cancer patients have shown that leptin levels are not elevated during weight loss demonstrating that the hormone leptin is not involved in the initiation of anorexia in this population [3].

A significant number of children who receive radiation or chemotherapy develop oral mucositis. Mucositis is the inflammation of mucosa as a result of ionizing radiation or chemotherapeutic drugs [21]. Lesions caused by mucositis can result in increased risk for systemic infection, significant pain and oral hemorrhage decreasing or inhibiting oral intake and increasing the risk for malnutrition (undernutrition). Frequent oral care is paramount to treatment. The medication palifermin (Kepivance^®^) was approved by the U.S. Food and Drug Administration for use in a subset of adult patients. Palifermin, the recombinant human keratinocyte growth factor is approved for adults with hematologic malignancies [22] to prevent or treat severe oral mucositis in those undergoing high dose chemotherapy, (with or without radiation) followed by HCST [22].

Hepatic veno-occlusive disease, also known as sinusoidal obstructive syndrome occurs with certain types of chemotherapy or after HSCT. It is a disease in which the blood vessels that flow within the liver and other surrounding organs supported by portal circulation are blocked. Veno-occlusive disease is the result of endothelial cell injury in the blood vessels adjacent to the liver and its ancillary organs induced by an increase in oxidative stress. The deficiency of the AOX glutathione (GSH) is believed to have a substantial role in the pathogenesis of this complication. A diet rich in AOX and adequate protein and energy may help reduce the frequency of this complication [10].

## 5. Dietary Supplements in Cancer

Complementary and Alternative Medicine, also known as CAM, is a group of diverse medical and health care products and practices that are not deemed part of conventional medicine [21,23]. CAM therapies are commonly used by the general public and consist of an enormous array of heterogeneous therapies that are for the most part not well understood by conventional healthcare providers [21]. CAM is challenging to define because of its range of therapies and the fact that its constituents are continuously being expanded. For example, a dietary supplement may consist of one or more dietary ingredients which include vitamins, minerals, herbs, amino acids, and other botanicals that are taken by mouth in the form of a capsules, liquids, pills or tablets [24]. The most frequently identified reasons for taking dietary supplements by individuals with cancer are to help cope with the adverse side effects of conventional treatment, to augment conventional anticancer therapy, and to prevent secondary malignancies [3,21,23]. It is estimated that between 35 and 50% of children with cancer in the U.S. take dietary supplements [21,23] and 6% to 91% use CAM [21,23]. 

Several supplements have been researched regarding their ability to modulate the progression of mucositis. Adverse effects of mucositis are associated with oral pain and difficulty swallowing, thereby increasing the risk of dehydration and/or malnutrition [21,25,26,27]. Traummel S^®^ (Heel Incorporated) is one such product. It is a homeopathic remedy made up of highly diluted botanical extracts and minerals. In a randomized controlled, double-blinded clinical trial involving 32 pediatric patients at multi-institutional sites it was shown to be effective in the reduction of stomatitis and mucositis in pediatric patients undergoing HSCT [21]. A subsequent international multi-center double-blinded, randomized study involving 181 patients with a much larger age range (3–25 years) undergoing HSCT did not confirm Traummel S^®^ as an effective treatment for mucositis. The latter study did find a trend toward less narcotic use for mouth pain [26] with the use of Traummel S^®^. Another supplement that is used to prevent and/or reduce the severity and/or duration of mucositis is glutamine (GLN) in children undergoing HSCT [21]. Glutamine is the most abundant amino acid in the plasma and muscles of humans [27]. Glutamine has several roles and is a precursor of glutamate, which is required for synthesis of the major AOX GSH [27]. One possible drawback to parenteral GLN use is that it requires a substantial amount of fluid administration [21], which may be contraindicated in children undergoing HSCT requiring fluid restriction [27].

## 6. Antioxidant Supplements in Cancer

Patients in the conditioning phase of treatment have been found to have reduced or depleted levels of AOX especially vitamin C [3,10], selenium, and vitamin E [28]. Several cancer treatments cause oxidative stress (an imbalance of the body’s pro-oxidants and AOX) as a part of their method of action to destroy cancer cells. The opposing states (low AOX level in conditioning versus oxidative stress mechanism of therapy) have prompted controversy regarding the benefit or harm of supplemental AOX at levels well beyond the recommended daily intake during cancer therapy [3,11,29]. These therapies exert a portion of their anticancer effects through the formation of free radicals also termed reactive oxygen species (ROS) [11,21,30]. Current U.S. Food and Drug Administration approved anticancer therapies that generate ROS include anthracyclines, arsenic trioxide, cytarabine, and vincristine [11]. The concern is that AOX could impair the action of particular types of anticancer therapy. The impairment of anticancer therapy with AOX use has not been confirmed by clinical trials [21]. Not all anticancer agents are involved in the formation of ROS as part of the anticancer effects. Thus, there is the potential benefit for AOX supplementation to improve the patient’s nutritional status and reduce toxicities related to chemotherapy in a subset of patients, though study results have been inconsistent [4,30].

The production of ROS is a normal aerobic physiological process that results from both endogenous and exogenous sources [31]. Examples of endogenous sources include activation of inflammatory cells, mitochondria metabolism, and peroxisomes, while exogenous sources include chemicals (industrial), environmental and pharmaceutical agents [30,31]. ROS have both a positive and negative function in cell proliferation and cell survival [11].

Antioxidants can also be broadly classified as enzymatic and non-enzymatic [30]. Examples of enzymatic AOX include superoxide dismutase, catalase dismutase, and glutathione peroxidase [32]. Non-enzymatic AOX are classified as metabolic or nutrient. Glutathione is a metabolic non-enzymatic AOX and vitamin C and E, carotenoids, selenium and thiol are classified as non-enzymatic nutrient AOX [32,33,34].

The normal balance between the production of ROS and AOX activity is maintained by the body in equilibrium. The loss of this balance is particularly important in leukemia cells. Leukemia cells inherently contain moderately elevated levels of ROS [11,31] as a consequence of alterations that occur in pro-oxidant and AOX pathways [11]. Pro-oxidant and AOX pathways potentially contribute to the susceptibility to cancer treatment in leukemia. Once ROS levels exceed that manageable by cells, oxidative damage ensues leading to lipid peroxidation and deoxyribonucleic acid damage [11,31]. The sustainment of higher ROS levels occurs in all four forms of leukemia. The higher ROS levels promote leukemogenesis and ultimately promote growth and survival of cancer cells even with insults from traditional cancer treatment. Leukemic cells frequently are dysregulated in both the expression and activity of diverse AOX pathways. In leukemia, the alteration in the oxidative balance plays a significant role in the survival, growth, progress and resistance to therapy. The oxidant balance in leukemia has an advantage toward ROS production. Reactive oxygen species producing complexes have the potential for significant clinical application; either by causing direct damage to cancer cells or through inhibition of growth and survival of potential cancer cells. For the most part, AOX are down-regulated in leukemia cells and thereby promote ROS signaling and genomic instability. However, upon treatment with ROS-producing agents, many AOX enzymes have the potential to be up-regulated and potentially promote resistance to anticancer treatment. Enhanced understanding of the oxidative balance in leukemia will lead to further advancement in the treatment of leukemia and reduction in the associated toxicities [11] and lead to evidence based nutritional guidelines for the use or non-use of AOX supplementation during and after anticancer therapy for pediatric leukemia.

## 7. Methods of Review

References were obtained using the search engine PubMed. Review of research articles and textbook references were limited to the years of publication from 2000 to present. The primary search criteria focused on pediatric leukemia, AOX assessment and/or measurement in this population. The search target was acute and long-term nutritional implications of pediatric ALL and its primary treatment modalities.

Pediatric patients are inherently different than adult patients. Pediatrics is categorized into several different age groups (neonates, infants, young children, and adolescents). Each group is at a different developmental and growth stage with different nutritional and energy needs [15]; thus children are not just small adults. Pediatric studies related to nutrition are generally small and somewhat sparse. Since the treatment modalities for pediatric ALL are more aggressive than in adults, and children have a higher survival rate, extrapolations of study results from adults to children are not appropriate. The available pediatric literature at this time shows a lack of consensus in criteria to identify malnutrition, and highlights the overall complexity of nutrition assessments in the pediatric leukemia population. A wide range of AOX levels were measured and/or evaluated as potential adjunct therapies in leukemic patients. Two studies specifically focused on mucositis therapy following anticancer treatment. An overview of research studies is listed by category and publication date in Table 1, Table 2 and Table 3.

**Table 1:** Overview of nutrition-related pediatric oncology studies—mucositis and nutrition care practices.

Author	Overview	Results
Srinivason, A. *et al.* 2012 [22]	Phase I cohort study Involved 12 children (median age 9.2 years), five males, seven females with ALL, and AML Assessment of tolerance to Palifermin in dose progression levels of 40, 60 and 90 μg/kg/day to assess toxicity risk	No evidence of toxicity with dosing 90 μg/kg/day based on pharmacokinetics: Most frequent side effect: macular rash Drug elimination occurred within 24–48 h of administration
Sencer, S.F. *et al.* 2012 [26]	International multi-institutional, double-blinded, randomized trial A total of 181 participants between the ages of 3–25 years; evaluation of the effectiveness of Traumeel S^®^ in the treatment of mucositis	Lack of confirmation of Traumeel S^®^: As an effective treatment for CT-induced mucositis Found trend toward reduced narcotic used
Ladas, E.J. *et al.* 2006 [35]	A total of 223 COG institutions were invited to complete a survey as a means to ascertain the degree of consensus practices utilized in assessment of nutritional status; either a MD, RD or RN completed the survey	A total of 120 COG facilities responded and it was found that nutritional assessments were performed using numerous different indices to: Identify nutritional status Classify malnutrition and its severity
Thornley, I. *et al.* 2004 [36]	Pilot Study, 37 participants Assessment of the benefit of the AOX: ursodexycholic acid, vitamin E, and folinic acid in children receiving PN undergoing HSCT	The most distinct benefits were found in high risk patients assessed by: Shorter time period to engraftment Reduced severity and incidence of mucositis Decreased severity of liver toxicity
Oberbaum, M. *et al.* 2001 [25]	A randomized, controlled clinical trial, 32 participants, ages 3–25 years, evaluation of TRAUMMEL S^®^ in the treatment of mucositis WHO grading score used to assess the severity of mucositis	TRAUMEEL S^®^ may have significantly lowered: Severity and duration of oral mucositis induced by SCT Mean mucositis days for placebo group 24.3; 10.4 for treatment group

^®^HEEL Corporation SCT/HSCT: stem cell transplantation/hematopoietic stem cell transplant; CT: chemotherapy; ALL: acute lymphoblastic leukemia; AML: acute myeloid leukemia; PN: parenteral nutrition; WHO: World Health Organization.

**Table 2:** Overview of nutrition-related pediatric oncology—studies of body composition.

Author	Overview	Results	
Karaman, S. *et al.* 2010 [37]	A total of 93 survivors of childhood ALL, 74 previously received CRT as part of ALL treatment as a child Fifty healthy adults similar in age served as controls The purpose of the study was to evaluate obesity risk factors (BMI and serum leptin levels) in adults treated with CRT for childhood ALL	Leptin and BMI levels: Levels for females who received CRT were significantly higher compared to the controls ALL treatment can lead to obesity	
Garmey, E.G. *et al.* 2008 [38]	Retrospective cohort study evaluating body composition (BMI) of 1451 survivors of childhood ALL over a 16-year period	ALL is associated with: A higher BMI in children that receive CRT during their first 10 years of life, particularly females	

ALL: acute lymphoblastic leukemia; CRT: cranial radiotherapy; BMI: body mass index.

**Table 3:** Overview of nutrition related pediatric oncology studies—antioxidants.

Author	Overview	Results
Radhakrishnan, N. *et al.* 2012 [28]	Case control study, 45 newly diagnosed children with ALL serum fasting levels of zinc, selenium, retinol and tocopherol were compared to an age-matched control group of 20	Patients with lower serum levels of selenium and tocopherol at diagnosis were found to be at greater risk for: Febrile neutropenia Sepsis during the first 8 weeks of therapy
Al-Tonbary, Y. *et al.* 2011 [39]	Prospective observational study involving Fifty newly diagnosed children with ALL between the ages of 1.5–12 years, median age 6.84 ± (SD) 3.73 years Oxidative stress (MDA, TAC) evaluated at diagnosis and completion of induction phase of CT Apoptosis evaluated at diagnosis and 1 week post treatment by fluoremetric TUNEL Healthy age and gender matched children served as controls	Compared to the controls, children with ALL at diagnosis and completion of induction had: Higher oxidative stress levels Low AOX levels Significantly elevated levels of apoptosis 1 week post induction phase compared to levels at diagnosis
Al-Tonbary, Y. *et al.* 2009 [40]	Cohort study, 40 participants NAC and vitamin E prescribed as adjuvant AOX therapy in pediatric ALL Twenty participants received vitamin E and NAC supplementation and 20 did not Levels of Glu.Px, MDA and TNF-α were obtained to evaluate AOX therapy	The vitamin E and NAC group were associated with a decrease in: Radiation and CT toxicities Blood and platelet transfusions Hepatic toxicities
Mazor, D. *et al.* 2008 [41]	Observational study, 13 children between the ages of 4–18 years with ALL or solid tumors evaluated AOX status and oxidative stress levels	Both groups had lower AOX and thiol levels The ALL group had substantially lower thiol levels There was a potentially higher oxidative stress level in the ALL group
Papageorgiou, M. *et al.* 2005 [42]	Observational study, 80 participants receiving CT, TAC and cTAC levels were evaluated	During CT: TAC and cTAC levels progressively declined TAC and cTAC levels remained low for 6 months post treatment
Aquino, V.M. *et al.* 2005 [43]	Double-blinded randomized placebo-controlled study,120 children Twenty-eight days following HSCT or hospital discharge in which 50% received oral-glycine and 50% received oral-GLN	The GLN group was found to havea reduction in the number of days requiring narcotics for mucositis GLN appears to be both safe and effective in decreasing the severity of mucositis
Kennedy, E. *et al.* 2004 [29]	Multi-centered, prospective, observational study of 103 children diagnosed with ALL The AOX levels of vitamin A, E, ascorbate, β-carotene, total carotenoid were evaluated Dietary intake of AOX: calculated based on 24 h Food recall and food frequency questionnaire obtained at three separate intervals	AOX intake was low for all except vitamin C Higher dietary intake of vitamin E at 3 months correlated with a lower infection rate Higher dietary intake of vitamin C and β-carotene at 6 months correlated with decreased toxicity related to therapy

ALL: acute lymphocytic leukemia; MDA: malondialdehyde; TAC: total AOX capacity; CT: chemotherapy; TUNEL: terminal deoxynucleotidyl transferase dUTP nick end labeling; AOX: antioxidants; GLN: glutamine; cTAC: corrected total; AOX capacity; NAC: *N*-acetylcysteine; Glu.Px: glutathione peroxidase; TNF-α: tumor necrosis factor alpha; HSCT: hematopoietic stem cell transplantation.

## 8. Future Directions

Research has shown that AOX levels are depleted in pediatric oncology patients receiving chemotherapy and/or radiation therapy. Antioxidants and ROS have dual biological functions in both the body’s normal physiological processes and in cancer development. Reactive oxygen species are generated as a byproduct of metabolism and in specific cancer treatments as a part of their anticancer mechanism, though not all anticancer treatments use ROS as a method to destroy cancer cells. Based on the few pediatric studies reviewed here, intake of AOX in this population is sub-optimal except for vitamin C. This population was associated with higher oxidative stress and low AOX levels. Specific AOX supplementation demonstrated a trend toward decreased therapeutic toxicity and severity of mucositis. The pediatric studies reviewed here have used an array of supplemental vitamin and mineral (AOX) cocktails in addition to diverse methods to evaluate both oxidative stress and study endpoints. Large randomized, double-blind trials are needed to ascertain the ideal dosage and optimal vitamin and mineral combinations of AOX to assess the benefit or harm in pediatric ALL patients.

## 9. Conclusions

It is well documented that the survival rate for pediatric ALL patients has approached 90%. However, current treatment modalities are associated with toxicities and an increased complexity in performing both nutrition assessments and interventions acutely and chronically. A unified criterion for identification of malnutrition or nutritional at risk pediatric oncology patients is greatly needed. Awareness of these complications has increased and evolving knowledge regarding oxidative stress and its impact on cancer development at the cellular and molecular level is in progress. This knowledge can be translated to the development of adjuvant therapeutic interventions to treat toxicities of cancer treatment or as cancer treatment modalities. Treatments of this nature should be given high priority in an effort to provide more effective cancer treatment with less toxicity and adverse nutritional related outcomes.
